# Supplementary material for: Predictive value of CD86 for the occurrence of sepsis (Sepsis-3) in patients with infection
Source: PLoS One. 2024 Apr 11;19(4):e0302063. doi: 10.1371/journal.pone.0302063 (PMC11008888; doi:10.1371/journal.pone.0302063)
Supplement: Attachment — Submitted filename: Responed-letter.docx [file pone.0302063.s003.docx]

**Title:** Predictive value of CD86 for the occurrence of sepsis (Sepsis-3) in patients with infection

**Journal:** PLoS ONE

**Response to Reviewers’ comments**

Dear Editor,

We thank you for your careful consideration of our manuscript. We appreciate your response and overall positive initial feedback and made modifications to improve the manuscript. After carefully reviewing the comments made by the Reviewers, we have reorganized the literature data, modified the manuscript to improve the presentation of our results and their discussion, therefore providing a complete context for the research that may be of interest to your readers.

We hope that you will find the revised paper suitable for publication, and we look forward to contributing to your journal. Please do not hesitate to contact us with other questions or concerns regarding the manuscript.

Best regards,

Keji Zhang

**Reviewer #1**

*The Manuscript needs some minor revisions:*

*-Improve the English form.*

**Response:** We thank the Reviewer. The manuscript was proofread.

*-It is unclear how control patients were enrolled. Do they always come from the emergency room? are they patients with fever? The authors should clarify the description of this group.*

**Response:** We thank the Reviewer for the comment. In this study, the patients who visited the emergency room were enrolled if they were ≥18 years of age and had a confirmed infection or were with a high suspicion of infection with clear clinical symptoms. The patients who were highly suspected of having an infection with clear clinical symptoms were also required to meet at least two of the following four diagnostic criteria for systemic inflammatory response syndrome [1]: 1) body temperature of >38℃ or <36℃, 2) heart rate of >90 beats/min, 3) respiratory rate of >20 breaths/min, or partial pressure of carbon dioxide of <32 mmHg, and 4) peripheral white blood cell count of >12,000/mm^3^ or <4000/mm^3^.

Then, all patients with confirmed infection or highly suspected of infection were divided into the sepsis and the non-sepsis groups according to the Sepsis-3 standard [2]. Finally, the patients with sepsis were divided into the sepsis-survival and sepsis-death groups according to whether the patient died or not. Therefore, there were no healthy controls, and all patients had infection (or were highly suspected of). It was clarified in the manuscript.

*- the tables and figures are clear except for figure 2, the symbols are not clear and are small, also the figure should be divided into A and B.*

**Response:** We thank the Reviewer for the comment. We agree that the group symbols were small and kind of lost in the line. Figure 2A represents the CD86%, HLA, and CD86 mRNA, while Figure 2B presents the flow cytometry outputs of CD86 among the three groups. It was revised accordingly.

**Reviewer #2**

*The authors have written a nice manuscript which is a prospective study.*

**Response:** We thank the Reviewer for his/her appreciation of our work.

1. *Variables with P<0.15 in the* *univariable regression analysis were selected for* *multivariable logistic regression analysis (MVA). Why not only variables with significant value p<0.05 were taken for MVA*

**Response:** We thank the Reviewer for the comment. Due to the effect of other covariates, some characteristics not significant in the univariable regression analyses may be significant in the multivariable regression. Therefore, we selected variables with P<0.15 in the univariable regression analysis. In addition, the sample size was relatively small, particularly the sepsis-death groups, and using a higher P-value decreases the risk of missing a variable that would be significant in the multivariable analysis. P-values thresholds as high as 0.25 have been reported [3-5]. Still, we agree that using a more stringent threshold could change the variables included in the multivariable models. The results will have to be confirmed in larger multicenter studies. It was added in the Discussion.

2. *Why were blood samples collected after 1st dose of* *antibiotics, and not at baseline?*

**Response:** We are deeply sorry for the error that occurred during manuscript preparation and translation. The blood samples were taken after admission and before the first dose of antibiotics. It was corrected.

3. *How can variables with such low odds ratios [PLT (OR=0.99, 95%CI: 0.986-0.997, P=0.001),* *IL-10 (OR=1.01)] be statistically significant? What is the outcome of odds ratio for these variables?*

**Response:** We thank the Reviewer for the comment. It is because the determination of the odds ratio is based on the change of one unit of PLT, for example. Therefore, for each change of 1×10^9^/L, the odds of sepsis decrease by 1%. Since the PLT levels are in the range of 91-316 ×10^9^/L, a change of 1×10^9^/L (which is small relative to the platelet levels) will lead to a small change in odds. It is a common observation with continuous variables in logistic regression models. For IL-10, the range was large, which could explain the results. In addition, in a multivariable analysis, each variable is adjusted for all the others [6-8].

4. *In Methods - Please write clearly what was the primary endpoint of the study. Writing to explore the value of CD86 and HLA-DR in sepsis, is very vague*

**Response:** We agree with the Reviewer. The primary study endpoint was the factors associated with a diagnosis of sepsis. The secondary study endpoint was the factors associated with mortality among patients with sepsis. It was clarified in the manuscript.

5. *How many patients who were initially classified as “non-sepsis” later turned into “sepsis” during the course of hospital stay? How were these patients accounted for?*

**Response:** The Reviewer raises a good point. Twenty-three patients changed from non-sepsis to sepsis during hospitalization. They were grouped in the sepsis group after meeting the diagnostic criteria for sepsis. It was clarified in the Methods and Results.

**Journal** **Requirements**

*When submitting your revision, we need you to address these additional requirements.*

*1. Please ensure that your manuscript meets PLOS ONE’s style requirements, including those for file naming. The PLOS ONE style templates can be found at*

*https://journals.plos.org/plosone/s/file?id=wjVg/PLOSOne_formatting_sample_main_body.pdf and*

*https://journals.plos.org/plosone/s/file?id=ba62/PLOSOne_formatting_sample_title_authors_affiliations.pdf*

**Response:** We revised the manuscript accordingly.

*2. We note that the grant information you provided in the ‘Funding Information’ and ‘Financial Disclosure’ sections do not match.*

*When you resubmit, please ensure that you provide the correct grant numbers for the awards you received for your study in the ‘Funding Information’ section.*

**Response:** It was verified.

*3. Thank you for stating the following in the Acknowledgments Section of your manuscript:*

*This work was supported by the Science and Technology Support Project for Medical Guidance (Chinese and Western Medicine) of Shanghai Municipal and the Science and Technology Commission in 2018 [No.18411967000]. The sponser had no role in study design, data collection and analysis, decision to publish, or preparation of the manuscript.*

**Response:** We are a little confused. The Journal asks here to add the funding information in the manuscript, but in the request just below, the Journal asks to remove all funding information from the manuscript. For now, we followed the instructions provided in point #1 and removed all funding statements from the manuscript.

*We note that you have provided funding information that is not currently declared in your Funding Statement. However, funding information should not appear in the Acknowledgments section or other areas of your manuscript. We will only publish funding information present in the Funding Statement section of the online submission form.*

*Please remove any funding-related text from the manuscript and let us know how you would like to update your Funding Statement. Currently, your Funding Statement reads as follows:*

*The funders had no role in study design, data collection and analysis, decision to publish, or preparation of the manuscript.*

*Please include your amended statements within your cover letter; we will change the online submission form on your behalf.*

**Response:** We follow the instructions provided in point #1 and removed all funding statements from the manuscript. The funders had no role in study design, data collection and analysis, decision to publish, or preparation of the manuscript.

*4. PLOS requires an* *ORCID iD for the corresponding author in Editorial Manager on papers submitted after December 6th, 2016. Please ensure that you have an ORCID iD and that it is validated in Editorial Manager. To do this, go to ‘Update my Information’ (in the upper left-hand corner of the main menu), and click on the Fetch/Validate link next to the ORCID field. This will take you to the ORCID site and allow you to create a new iD or authenticate a pre-existing iD in Editorial Manager. Please see the following video for instructions on linking an ORCID iD to your Editorial Manager account: https://www.youtube.com/watch?v=_xcclfuvtxQ*

**Response:** We are now providing the ORCID iD for the corresponding author.

*5. Your ethics statement should only appear in the Methods section of your manuscript. If your ethics statement is written in any section besides the Methods, please delete it from any other section.*

**Response:** It was revised.

*6. Please include captions for your Supporting Information files at the end of your manuscript, and update any in-text citations to match accordingly. Please see our Supporting Information guidelines for more information: http://journals.plos.org/plosone/s/supporting-information.*

**Response:** We do not provide any supporting information.

**References**

1.	Chakraborty RK, Burns B. Systemic Inflammatory Response Syndrome. StatPearls. Treasure Island (FL)2022.

2.	Sansom DM, Manzotti CN, Zheng Y. What’s the difference between CD80 and CD86? Trends Immunol. 2003;24(6):314-9. Epub 2003/06/18. doi: 10.1016/s1471-4906(03)00111-x. PubMed PMID: 12810107.

3.	Bursac Z, Gauss CH, Williams DK, Hosmer DW. Purposeful selection of variables in logistic regression. Source Code Biol Med. 2008;3:17. Epub 2008/12/18. doi: 10.1186/1751-0473-3-17. PubMed PMID: 19087314; PubMed Central PMCID: PMCPMC2633005.

4.	Sauerbrei W, Perperoglou A, Schmid M, Abrahamowicz M, Becher H, Binder H, et al. State of the art in selection of variables and functional forms in multivariable analysis-outstanding issues. Diagn Progn Res. 2020;4:3. Epub 2020/04/09. doi: 10.1186/s41512-020-00074-3. PubMed PMID: 32266321; PubMed Central PMCID: PMCPMC7114804.

5.	Chowdhury MZI, Turin TC. Variable selection strategies and its importance in clinical prediction modelling. Fam Med Community Health. 2020;8(1):e000262. Epub 2020/03/10. doi: 10.1136/fmch-2019-000262. PubMed PMID: 32148735; PubMed Central PMCID: PMCPMC7032893.

6.	Ranganathan P, Pramesh CS, Aggarwal R. Common pitfalls in statistical analysis: Logistic regression. Perspect Clin Res. 2017;8(3):148-51. Epub 2017/08/23. doi: 10.4103/picr.PICR_87_17. PubMed PMID: 28828311; PubMed Central PMCID: PMCPMC5543767.

7.	Roalfe AK, Holder RL, Wilson S. Standardisation of rates using logistic regression: a comparison with the direct method. BMC Health Serv Res. 2008;8:275. Epub 2008/12/31. doi: 10.1186/1472-6963-8-275. PubMed PMID: 19113996; PubMed Central PMCID: PMCPMC2661894.

8.	Stoltzfus JC. Logistic regression: a brief primer. Acad Emerg Med. 2011;18(10):1099-104. Epub 2011/10/15. doi: 10.1111/j.1553-2712.2011.01185.x. PubMed PMID: 21996075.
