# Supplementary material for: Predictive value of CD86 for the occurrence of sepsis (Sepsis-3) in patients with infection
Source: PLoS One. 2024 Apr 11;19(4):e0302063. doi: 10.1371/journal.pone.0302063 (PMC11008888; doi:10.1371/journal.pone.0302063)
Supplement: Attachment — Submitted filename: Response letter.docx [file pone.0302063.s004.docx]

**Title:** Predictive value of CD86 for the occurrence of sepsis (Sepsis-3) in patients with infection

**Journal:** PLOS ONE

**Response to Reviewers' comments**

Dear Editor,

We thank you for your careful consideration of our manuscript. We appreciate your response and overall positive initial feedback and made modifications to improve the manuscript. After carefully reviewing the comments made by the Reviewers, we have reorganized the literature data, modified the manuscript to improve the presentation of our results and their discussion, therefore providing a complete context for the research that may be of interest to your readers.

We hope that you will find the revised paper suitable for publication, and we look forward to contributing to your journal. Please do not hesitate to contact us with other questions or concerns regarding the manuscript.

Best regards,

Keji Zhang

**Academic Editor**

*Journal Requirements:*
*Please review your reference list to ensure that it is complete and correct. If you have cited papers that have been* *retracted, please include the rationale for doing so in the manuscript text, or remove these references and replace them with relevant current references. Any changes to the reference list should be mentioned in the rebuttal letter that accompanies your revised manuscript. If you need to cite a retracted article, indicate the article&rsquo;s retracted status in the References list and also include a citation and full reference for the retraction notice.*

**Response:** We thank the Academic Editor. We verified our references. We did not cite any references flagged as retracted on PubMed.

**Reviewer #3**

*1. If the authors have adequately addressed your comments raised in a previous round of review and you feel that this manuscript is now acceptable for publication, you may indicate that here to bypass the &ldquo;Comments to the Author&rdquo; section, enter your conflict of interest statement in the &ldquo;Confidential to Editor&rdquo; section, and submit your &quot;Accept&quot; recommendation.*

*Reviewer #3: (No Response)*

*2. Is the manuscript technically sound, and do the data support the conclusions?
The manuscript must describe a technically sound piece of scientific research with data that supports the conclusions. Experiments must have been conducted rigorously, with appropriate controls, replication, and sample sizes. The conclusions must be drawn appropriately based on the data presented.*

*Reviewer #3: Yes*

*3. Has the statistical analysis been performed appropriately and rigorously?*

*Reviewer #3: Yes*

*4. Have the authors made all data underlying the findings in their manuscript fully available?
The* [*PLOS Data policy*](http://www.plosone.org/static/policies.action#sharing) *requires authors to make all data underlying the findings described in their manuscript fully available without restriction, with rare exception (please refer to the Data Availability Statement in the manuscript PDF file). The data should be provided as part of the manuscript or its* *supporting information, or deposited to a public repository. For example, in addition to summary statistics, the data points behind means, medians and variance measures should be available. If there are restrictions on publicly sharing data&mdash;e.g. participant privacy or use of data from a third party&mdash;those must be specified.*

*Reviewer #3: No*

**Response:** We thank the Reviewer for the comment. As per local regulations in our hospital, we are not allowed to upload our data on public registries for now, but the data can be made available to researchers upon reasonable request. As a result, we are unable to provide the requested deposition at this time. We understand the importance of these processes and will continue to explore possibilities for compliance with such requirements in the future.

*5. Is the manuscript presented in an intelligible fashion and written in standard English?
PLOS ONE does not copyedit accepted manuscripts, so the language in submitted articles must be clear, correct, and unambiguous. Any typographical or grammatical errors should be corrected at revision, so please note any specific errors here.*

*Reviewer #3: Yes*

*6. Review Comments to the Author
Please use the space provided to explain your answers to the questions above. You may also include additional comments for the author, including concerns about dual publication, research ethics, or publication ethics. (Please upload your review as an attachment if it exceeds 20,000 characters)*

*Reviewer #3: The authors present a well performed observational study and the manuscript is well organized and written. The control group with infection and signs of systemic inflammation is well chosen and reflects the clinical question sepsis biomarkers are needed for.
To my understanding the data availability does not fulfill PLOS requirements. The data should either be available from a repository or you should explain why not. Apart from that the manuscript is suitable or publication.*
**Response:** We thank the Reviewer for the comment. As per local regulations in our hospital, we are not allowed to upload our data on public registries, but the data can be made available to researchers upon reasonable request. As a result, we are unable to provide the requested deposition at this time. We understand the importance of these processes and will continue to explore possibilities for compliance with such requirements in the future.

*Apart from that I have only minor remarks that might be considered by the author but are not relevant for the deission to publish. The discussion is a little bit lengthy reviewing a lot of molecular biology mainly of interest to a small section of the potentiall readership. Maybe citing a good review cold shorten it, but as mentioned this might just be my personell preference.*

**Response:** We thank the Reviewer for the comment. We agree with the Reviewer that this study did not examine the exact molecular biology and that the discussion, even if explanatory, might be superfluous to many of the readership. Therefore, as suggested, we cited good reviews, but kept the discussion considering that it explains our results. Hence, the interested readership will be able to seek more information.

*In table one I am not sure if you relly should test for significant differences in septic shock, ventialition or SOFA scores. All these variables ae hallmarks of organ failure and therefore the fulfillment of sepsis-3 criteria and the differences between the sepsis and non-sepsis group are therefore not random by design.*

**Response:** We understand the Reviewer’s comment. We simply wanted to show the characteristics of the patients.
